# Supplementary material for: Observation of vortex-string chiral modes in metamaterials
Source: Nat Commun. 2024 Mar 14;15:2332. doi: 10.1038/s41467-024-46641-w (PMC10940314; doi:10.1038/s41467-024-46641-w)
Supplement: Supplementary file 1 — Supplementary Information [file 41467_2024_46641_MOESM1_ESM.pdf]

Supplementary Notes of

Observation of vortex-string chiral modes in metamaterials

Jingwen Ma[1], Ding Jia[2,3], Li Zhang[2,4,5,6], Yi-jun Guan[3], Yong Ge[3], Hong-xiang Sun[3,7,*], Shou-qi Yuan[3], Hongsheng Chen[2,4,5,6], Yihao Yang[2,4,5,6,*], Xiang Zhang[1,*]

[1]Faculties of Science and Engineering, The University of Hong Kong, Hong Kong, China

[2]Interdisciplinary Center for Quantum Information, State Key Laboratory of Extreme Photonics and Instrumentation, ZJU-Hangzhou Global Scientific and Technological Innovation Center, Zhejiang University, Hangzhou 310027, China

[3]Research Center of Fluid Machinery Engineering and Technology, School of Physics and Electronic Engineering, Jiangsu University, Zhenjiang 212013, China

[4]International Joint Innovation Center, The Electromagnetics Academy at Zhejiang University, Zhejiang University, Haining 314400, China

[5]Key Laboratory of Advanced Micro/Nano Electronic Devices & Smart Systems of Zhejiang, Jinhua Institute of Zhejiang University, Zhejiang University, Jinhua 321099, China

[6]Shaoxing Institute of Zhejiang University, Zhejiang University, Shaoxing 312000, China

[7]State Key Laboratory of Acoustics, Institute of Acoustics, Chinese Academy of Sciences, Beijing 100190, China

[*]Correspondence to: (Y. Y.) yangyihao@zju.edu.cn; (H. -X. S.) jsdxshx@ujs.edu.cn; (X. Z.) president@hku.hk

1. Design and theoretical analysis

Metamaterials, in general, are systematically designed macroscopic structures engineered to have specific responses to external excitations. These responses can be tailored and unconventional, enabling metamaterials to possess properties unattainable in conventional materials made up of atomic-scale unit cells. Metamaterials are typically based on periodic arrays of mesoscale artificial unit cells. These unique properties are typically derived from periodic arrays of mesoscale artificial unit cells, with their specific shape, size, and geometric orientation determining the desired macroscopic characteristics. Metamaterials provide a means to explore astronomical phenomena in controlled laboratory settings. For example, they have been used to simulate celestial mechanics[S1]. Over recent decades, metamaterials have also been employed to realize various topological charges predicted in high-energy physics, including Weyl[S2], Dirac[S3], and Yang monopoles[S4]. This section will focus on the rational design of a phononic metamaterial that exhibits a 5D Yang-monopole Hamiltonian.

1.1. Two-dimensional phononic graphene-like metamaterials

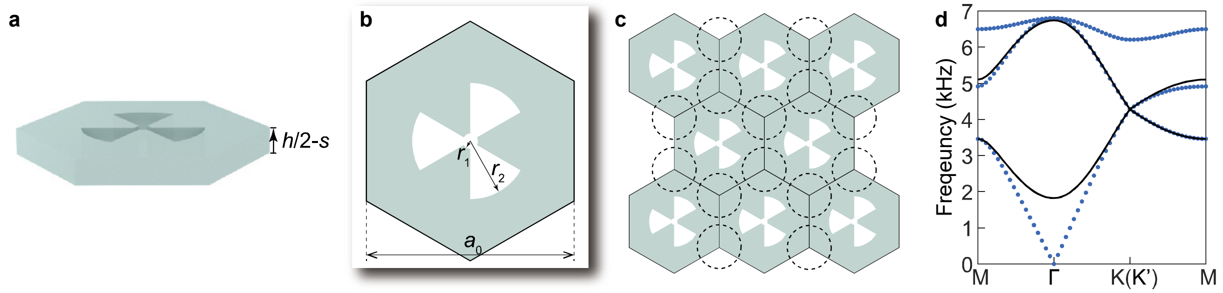

Supplementary Fig. 1 | 2D phononic graphene-like metamaterial. a, Unit-cell structure of a 2D phononic metamaterial containing a single planar layer with fan-shaped scatters. The layer thickness is $h/2 - s$, where $h = 32$ mm and $s = 11$ mm. **b, c**, Top view of the metamaterial exhibiting graphene lattice with lattice constants of $a_0 = 41$ mm. The fan-shaped scatters have dimensions of $r_1 = 1.5$ mm and $r_2 = 11.5$ mm. **d,** Band diagrams of graphene phononic metamaterial. The analytical results (black lines) obtained using tight-binding approximations agree well with the numerical results (blue dots) from commercial simulation software, confirming that theoretical analysis based on tight-binding approximation is sufficiently accurate for describing the physics near the degenerate point frequency ω_{YM}.

We initially focus on a simple 2D structure consisting of a single planar layer with fan-shape scatters. As shown in Figs. S1a–c, the fan-shape scatters predominantly confine acoustic waves within the sub-lattices, as indicated by the dashed circles in Supplementary Fig. 1c. We use tight-

binding approximation to theoretically study the physics of this metamaterial. Tight-binding approximation has been initially used in solid-state physics to calculate electronic band structures. It assumes that electrons are tightly confined to each lattice sites and can only slightly hop to nearby lattices to form band structures. Similarly, we assume that each sub-lattice exhibits on-site resonant frequency of ω_{YM}, and the nearest-neighbor hopping rate t_c. Consequently, the Hamiltonian for the 2D graphene metamaterial is

$$H\left(\vec{k}\right)\begin{pmatrix} u_1 \\ u_2 \end{pmatrix} = \begin{bmatrix} \omega_{\mathrm{YM}} & -\beta\left(\vec{k}\right) \\ -\beta^*\left(\vec{k}\right) & \omega_{\mathrm{YM}} \end{bmatrix}\begin{pmatrix} u_1 \\ u_2 \end{pmatrix}, \tag{S1}$$

where $\beta(\vec{k}) = t_n\left[\exp\left(-i\frac{\sqrt{3}k_2a_0}{3}\right) + 2\cos\left(\frac{k_1a_0}{2}\right)\exp\left(i\frac{\sqrt{3}k_2a_0}{6}\right)\right]$ is the nearest hopping between two sub-lattices. The sub-lattice weights of Bloch waves are represented by u_1 and u_2. The eigenvalues can be readily obtained as $\omega_{\mathrm{YM}} \pm \left|\beta(\vec{k})\right|$. Figure S1d shows the phononic band diagrams calculated using the commercial finite-element software COMSOL (blue dots), which agree well with the analytical values of $\omega_{\mathrm{YM}} \pm \left|\beta(\vec{k})\right|$ (black lines in Fig. S1d) near the Dirac-point frequency with the fitted value of nearest hopping term t_n = 0.82 kHz and on-site frequency ω_{YM} = 4.283 kHz.

1.2. Three-dimensional phononic Weyl-monopole metamaterials

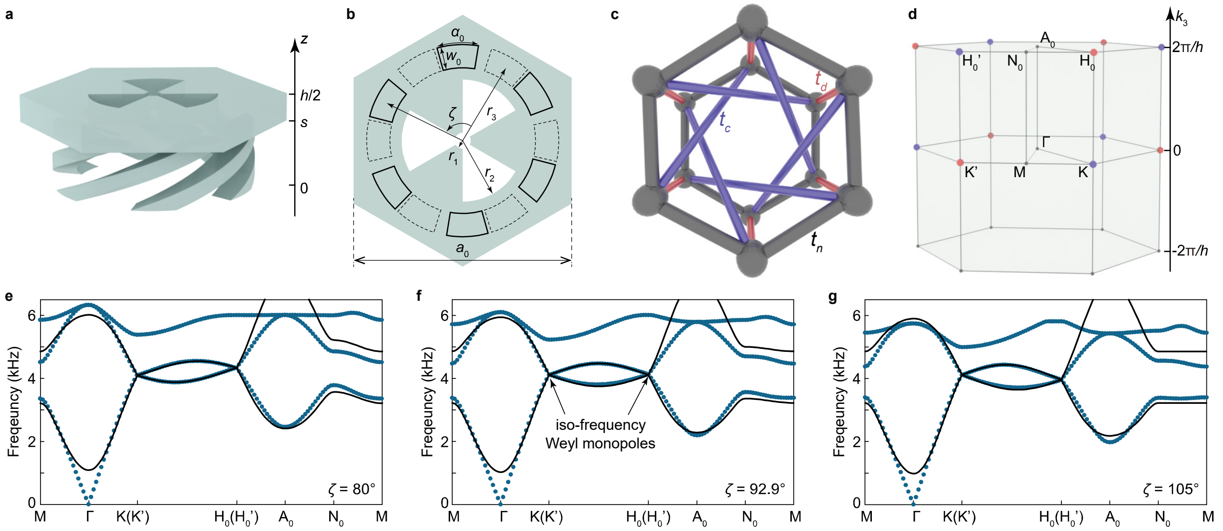

Supplementary Fig. 2 | 3D phononic Weyl-monopole metamaterial. a, Unit-cell structure of a 3D phononic Weyl-monopole metamaterial. Each unit cell contains six spiral air channels

connecting the planar air layers with fan-shape scatters. The lattice constants in the *x-y* plane and in the *z*-direction are $a_0 = 41$ mm and $h/2 = 16$ mm, respectively. The height of the spiral channels in the *z*-direction is $s = 11$ mm. The geometry of the plane air layer is exactly same to that shown in Supplementary Fig. 1. **b,** Sectional view of geometry at cutting planes $z = s$. The spiral air channels twist from $z = 0$ (dashed annulus sectors) to $z = s$ (solid annulus sectors) plane with a twist angle $\zeta = 92.9°$. The spiral air channels have dimensions of $\alpha_0 = 25°$ and $w_0 = 4.375$ mm with radius $r_3 = 15.875$ mm, and the fan-shape scatters in the planar air layers have dimensions of $r_1 = 1.5$ mm and $r_2 = 11.5$ mm. **c,** Illustration of tight-binding model of the structure. The intralayer nearest hopping rate is t_n (noted by black bond), the direct interlayer coupling rate is t_d (noted by red bond), and the chiral interlayer coupling rate is t_c (noted by blue bond). **d,** Illustration of first Brillouin zone in 3D momentum space. The range of the first Brillouin zone along the *z*-direction is $-2\pi/h \leq k_z \leq 2\pi/h$. The high-symmetry points K(K') and H_0(H_0') support Weyl points with topological charge $C_1 = 1$ (red point) or -1(blue point). **e–g,** Numerically calculated band diagrams of phononic metamaterials with (**e**) $\zeta = 80°$, (**f**) $\zeta = 92.9°$, (**g**) $\zeta = 105°$. Black lines represent theoretically calculated band diagrams under tight-binding approximation.

We proceed to investigate 3D metamaterials comprising multiple layers of 2D graphene structures discussed in Supplementary Fig. 1. The geometry of such 3D metamaterial is shown in Figs. S2a and b. The planar layers are connected by spiral channels along the *z* direction. In tight-binding approximation, the spiral channels introduce nonzero values of chiral interlayer coupling t_c (blue bond in Supplementary Fig. 2c) and direct interlayer coupling t_d (red bond in Supplementary Fig. 2c). The twist angle of the spiral channels ζ controls the relative values of t_c and t_d.

As this structure is periodic along the *z* direction, k_3 is a good quantum number. For each fixed k_3, and if we consider the dispersion and transport in the *x–y* plane, the Hamiltonian under tight-binding approximation is:

$$H(\vec{k})\begin{pmatrix} u_1 \\ u_2 \end{pmatrix} = \begin{bmatrix} \gamma(\vec{k}) - \alpha(\vec{k}) & -\beta(\vec{k}) \\ -\beta^*(\vec{k}) & \gamma(\vec{k}) + \alpha(\vec{k}) \end{bmatrix} \begin{pmatrix} u_1 \\ u_2 \end{pmatrix} \tag{S2}$$

with

$$\begin{aligned} \gamma(\vec{k}) &= \omega_{\text{YM}} - 2\cos(k_3 h/2)\left[t_d + t_c \cos(k_1 a_0) + 2t_c \cos\left(\sqrt{3}k_2 a_0/2\right)\cos(k_1 a_0/2)\right], \\ \alpha(\vec{k}) &= 2t_c \sin(k_3 h/2)\left[\sin(k_1 a_0) - 2\cos\left(\sqrt{3}k_2 a_0/2\right)\sin(k_1 a_0/2)\right], \\ \beta(\vec{k}) &= t_n\left[\exp\left(-i\sqrt{3}k_2 a_0/3\right) + 2\cos(k_1 a_0/2)\exp\left(i\sqrt{3}k_2 a_0/6\right)\right]. \end{aligned}$$

This Hamiltonian is similar to the 2D graphene Hamiltonian in Eq. (S1), except that the diagonal components are modified by the interlayer coupling terms caused by nonzero values of t_d and t_c.

The eigenvalues of the Hamiltonian in Eq. (S2) are $\gamma(\vec{k}) \pm \sqrt{\alpha(\vec{k})^2 + |\beta(\vec{k})|^2}$, so they features band-touching Weyl points when $\alpha(\vec{k}) = \beta(\vec{k}) = 0$. This is satisfied at specific points K = $\left(\frac{4\pi}{3a}, 0, 0\right)$, K' = $\left(\frac{-4\pi}{3a}, 0, 0\right)$, $H_0 = \left(\frac{4\pi}{3a}, 0, \frac{2\pi}{h}\right)$, and $H'_0 = \left(\frac{-4\pi}{3a}, 0, \frac{2\pi}{h}\right)$. The term $\gamma(\vec{k})$ can effectively tilt the conical dispersion along the z direction. The Weyl points at K (K') and H_0 (H'_0) are iso-frequency only when $2t_d = 3t_c$. Figure S3e–f shows the band diagrams of 3D Weyl-monopole metamaterials with different values of ζ = 80°, 92.9°, and 105° respectively. The theoretical results based on tight-binding approximations (black lines) agree well with the simulated results using COMSOL (blue dots), with the fitted parameters $(\omega_{\mathrm{YM}}, t_n, t_d, t_c) =$ (4.22, 0.82, 0.15, 0.06) kHz for Supplementary Fig. 2e, $(\omega_{\mathrm{YM}}, t_n, t_d, t_c) =$ (4.12, 0.82, 0.105, 0.07) kHz for Supplementary Fig. 2f, and $(\omega_{\mathrm{YM}}, t_n, t_d, t_c) =$ (4.045, 0.82, 0.075, 0.075) kHz for Supplementary Fig. 2g.

For the specific case of ζ = 92.9° (Supplementary Fig. 2f), one can conduct Taylor expansion around the K(K') and H_0 (H'_0) points to obtain the effective Weyl Hamiltonians

$$\begin{cases} H_{\mathrm{K/K'}}\left(\vec{k}\right) = \omega_{\mathrm{YM}} \pm v_1\left(k_1 \mp \dfrac{4\pi}{3a}\right)\boldsymbol{\sigma}_x - v_2 k_2 \boldsymbol{\sigma}_y + v_3 k_3 \boldsymbol{\sigma}_z \\ H_{\mathrm{H_0/H'_0}}\left(\vec{k}\right) = \omega_{\mathrm{YM}} \pm v_1\left(k_1 \mp \dfrac{4\pi}{3a}\right)\boldsymbol{\sigma}_x - v_2 k_2 \boldsymbol{\sigma}_y - v_3\left(k_3 - \dfrac{2\pi}{h}\right)\boldsymbol{\sigma}_z \end{cases},$$

where $v_1 = v_2 = \sqrt{3}t_n a_0/2$, $v_3 = 3\sqrt{3}t_c h/2$ are the Fermi velocity along x, y, and z directions. The theoretical analysis in this section clearly confirmed that the structures in Supplementary Fig. 2 can support Weyl monopoles at K(K') and H_0 (H'_0) points of the Brillouin zone.

1.3. Five-dimensional phononic Yang-monopole metamaterials

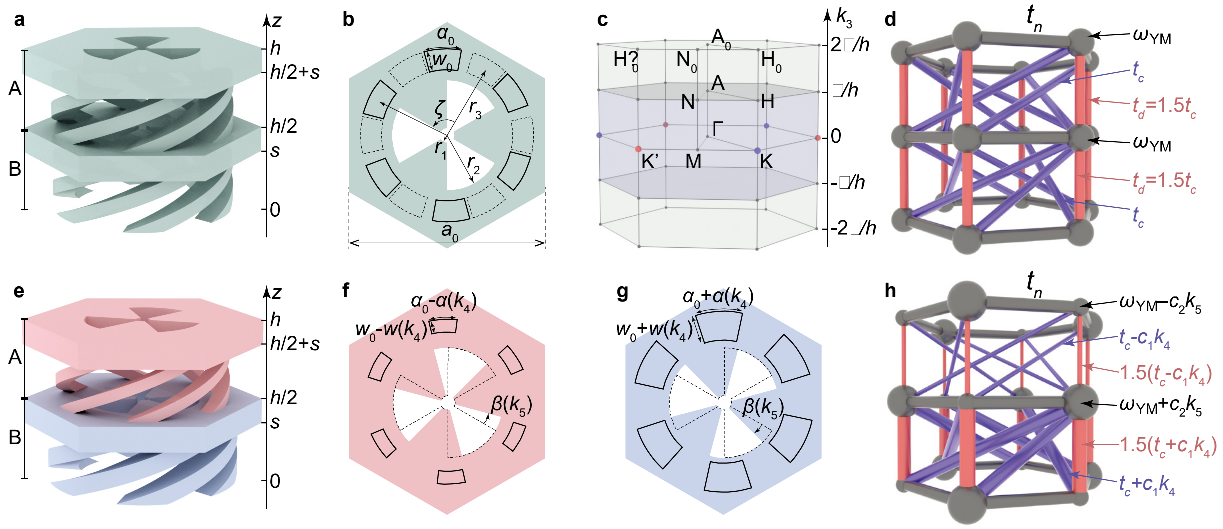

Supplementary Fig. 3 | Phononic Yang-monopole metamaterial. a, Unit-cell structure of the phononic metamaterial preserving half-lattice translational symmetry along *z* direction. Each unit cell contains two substructures A and B with the same geometry, and each substructure contains six spiral air channels connecting the plane air layers with fan-shape scatters. The lattice constants in the *x*-*y* plane and in the *z*-direction are a_0 = 41 mm and h = 32 mm, respectively. The height of the spiral channels in the *z*-direction is s = 11 mm. **b,** Sectional view of geometry at cutting planes $z = s$ and $z = h/2 + s$. The spiral air channels twist from $z = 0$ (dashed annulus sectors) to $z = s$ (solid annulus sectors) plane with a twist angle $\zeta = 92.9°$. The spiral air channels have dimensions of $\alpha_0 = 25°$ and w_0 = 4.375 mm with radius r_3 = 15.875 mm, and the fan-shape scatters in the plane air layers have dimensions of r_1 = 1.5 mm and r_2 = 11.5 mm. **c,** Illustration of first Brillouin zone in 3D momentum space. When the half-lattice translational symmetry is preserved, the minimum lattice constant along the *z*-direction is $h/2$, so that the range of the first Brillouin zone along the *z*-direction is $-2\pi/h \leqslant k_3 \leqslant 2\pi/h$. Breaking the half-lattice translational symmetry folds the first Brillouin zone to the region $-\pi/h \leqslant k_3 \leqslant \pi/h$. **d,** Illustration of tight-binding model of the structure preserving half-lattice translational symmetry. The intralayer nearest hopping rate is t_n (black bond), the chiral interlayer coupling rate is t_c (blue bond), and the direct interlayer coupling rate is $t_d = 1.5t_c$ (red bond). On-site frequency of each sublattice is ω_{YM}. **e,** Unit cell structure of the phononic metamaterial with broken half-lattice translational symmetry along the *z* direction. The substructures A and B (marked in red and blue) have different geometries controlled by two synthetic parameters (k_4, k_5). **f, g,** Sectional view of geometry at cutting planes $z = h/2 + s$ (**f**) and $z = s$ (**g**). In the substructure A (B), the parameter k_4 shrinks (expands) the size of the spiral air channels to $\alpha_0 \mp \alpha(k_4)$ and $w_0 \mp w(k_4)$ with $\alpha(k_4) = k_4 \cdot$ 7.5° and $w(k_4) = k_4 \cdot$ 2.375 mm, and k_5 rotates the fan-shape scatters counterclockwise (clockwise) by $\beta(k_5) = \mathrm{asin}(k_5)/3$ in radian unit. **h,** Illustration of tight-binding model of the structure with broken half-lattice translational symmetry. Nonzero k_4 and k_5 modify the on-site resonant frequency and interlayer coupling rate respectively.

In this section, we will use a band-folding technique to construct Yang-monopole metamaterials from the Weyl-monopole metamaterials discussed in Sec. 1.2. The structure of the Yang-

monopole metamaterial is shown in Supplementary Fig. 3a. It contains two substructures A ($0 < z < h/2$) and B ($h/2 < z < h$) with the same geometry described in Supplementary Fig. 2a, so that the entire structure has a half-lattice translational symmetry along the z direction. Such structures can be described by the tight-binding model shown in Supplementary Fig. 3d.

We further consider the unit-cell structures with broken half-lattice translational symmetry along the z direction (Supplementary Fig. 3e). Specifically, Figs. S3f and g show the geometry at cutting planes $z = s$ and $z = h/2 + s$, respectively, suggesting that the substructures A and B (marked in red and blue in Supplementary Fig. 3e) have different geometries defined by parameters (k_4, k_5), where k_4 shrinks (expands) the size of the spiral air channels to $\alpha_0 \mp \alpha(k_4)$ and $w_0 \mp w(k_4)$ with $\alpha(k_4) = k_4 \cdot 7.5°$ and $w(k_4) = k_4 \cdot 2.375$ mm, and k_5 rotates the fan-shape scatters counterclockwise (clockwise) by $\beta(k_5) = \mathrm{asin}(k_5)/3$ in radian unit. As shown in Supplementary Fig. 3h, in the tight binding model, nonzero k_4 modifies the direct and chiral interlayer coupling rates to $3(t_c \pm c_1 k_4)/2$ and $t_c \pm c_1 k_4$, and nonzero k_5 modifies the onsite frequency to $\omega_{\mathrm{YM}} \pm c_2 k_5$. The Hamiltonian is

$$H(\vec{k})\begin{pmatrix} u_1 \\ u_2 \\ u_3 \\ u_4 \end{pmatrix} = \begin{bmatrix} \omega_{\mathrm{YM}} + c_2 k_5 & -\beta(\vec{k}) & -\gamma_1(\vec{k}) + i\alpha_1(\vec{k}) & 0 \\ -\beta^*(\vec{k}) & \omega_{\mathrm{YM}} - c_2 k_5 & 0 & -\gamma_2(\vec{k}) + i\alpha_2(\vec{k}) \\ -\gamma_1(\vec{k}) - i\alpha_1(\vec{k}) & 0 & \omega_{\mathrm{YM}} - c_2 k_5 & -\beta(\vec{k}) \\ 0 & -\gamma_2(\vec{k}) - i\alpha_2(\vec{k}) & -\beta^*(\vec{k}) & \omega_{\mathrm{YM}} + c_2 k_5 \end{bmatrix} \begin{pmatrix} u_1 \\ u_2 \\ u_3 \\ u_4 \end{pmatrix} \quad \text{(S3)}$$

with

$$\begin{aligned}
\gamma_1(\vec{k}) &= t_c\left[3\cos(k_3 h/2) + 2\cos(k_3 h/2 + k_1 a_0) + 4\cos(k_3 h/2 - k_1 a_0/2)\cos\left(\sqrt{3}k_2 a_0/2\right)\right], \\
\alpha_1(\vec{k}) &= c_1 k_4\left[3\sin(k_3 h/2) + 2\sin(k_3 h/2 + k_1 a_0) + 4\sin(k_3 h/2 - k_1 a_0/2)\cos\left(\sqrt{3}k_2 a_0/2\right)\right], \\
\gamma_2(\vec{k}) &= t_c\left[3\cos(k_3 h/2) + 2\cos(k_3 h/2 - k_1 a_0) + 4\cos(k_3 h/2 + k_1 a_0/2)\cos\left(\sqrt{3}k_2 a_0/2\right)\right] \\
\alpha_2(\vec{k}) &= c_1 k_4\left[3\sin(k_3 h/2) + 2\sin(k_3 h/2 - k_1 a_0) + 4\sin(k_3 h/2 + k_1 a_0/2)\cos\left(\sqrt{3}k_2 a_0/2\right)\right], \\
\beta(\vec{k}) &= t_n\left[\exp\left(-i\sqrt{3}k_2 a_0/3\right) + 2\cos(k_1 a_0/2)\exp\left(i\sqrt{3}k_2 a_0/6\right)\right].
\end{aligned}$$

Near the YM momentum $\vec{k}_{\rm YM} = \left(k_1^{\rm YM}, k_2^{\rm YM}, k_3^{\rm YM}, k_4^{\rm YM}, k_5^{\rm YM}\right) = (4\pi m/3a_0\,, 0, 0, 0, 0)$ where $m =$ ±1 represent the YM at K and K' points respectively. The above Hamiltonian can be Taylor expanded into the following form:

$$H_{\rm YM} = \omega_{\rm YM}\mathbf{I} + \sum_{n=1}^{5} v_n \left(k_n - k_n^{\rm YM}\right)\mathbf{\Gamma}_n, \tag{S4}$$

where $(v_1, v_2, v_3, v_4, v_5) = \left(\sqrt{3}a_0 t_n m/2\,,\ \sqrt{3}a_0 t_n/2\,,\ 3\sqrt{3}ht_c m/2\,, 3\sqrt{3}c_1 m,\ c_2\right)$ is the Fermi velocity along five dimensions. The five 4×4 Gamma matrices $(\mathbf{\Gamma}_1, \mathbf{\Gamma}_2, \mathbf{\Gamma}_3, \mathbf{\Gamma}_4, \mathbf{\Gamma}_5) = \left(\boldsymbol{\sigma}_x, -\boldsymbol{\sigma}_y,\ -\boldsymbol{\sigma}_z\boldsymbol{\tau}_x,\right.$ $\left.\boldsymbol{\sigma}_z\boldsymbol{\tau}_y, \boldsymbol{\sigma}_z\boldsymbol{\tau}_z\right)$. Since we mainly focus on physics near YM, in the following discussion, we simply denote $k_n - k_n^{\rm YM}$ (the relative momentum to the position of YM) as k_n. By applying unitary transformations $\mathbf{U} = \left(\mathbf{I} - i\boldsymbol{\tau}_y\right)/\sqrt{2}$ and absorbing the anisotropy of v_n (n = 1–5) into the momentum term $\vec{k} = (k_1, k_2, k_3, k_4, k_5)$, we can rearrange the Hamiltonian into the following form:

$$H_{\rm YM} = \omega_{\rm YM}\mathbf{I} + v_D \sum_{n=1}^{5} k_n \mathbf{\Gamma}_n, \tag{S5}$$

where the five 4×4 Gamma matrices $(\mathbf{\Gamma}_1, \mathbf{\Gamma}_2, \mathbf{\Gamma}_3, \mathbf{\Gamma}_4, \mathbf{\Gamma}_5) = \left(\boldsymbol{\sigma}_x, -\boldsymbol{\sigma}_y, \boldsymbol{\sigma}_z\boldsymbol{\tau}_z, \boldsymbol{\sigma}_z\boldsymbol{\tau}_y, \boldsymbol{\sigma}_z\boldsymbol{\tau}_x\right)$ is slightly different from Eq. (S4) due to the unitary transformation. This Hamiltonian operates on the vector $\vec{u}_m = \left(u_m^{+,\uparrow}, u_m^{+,\downarrow}, u_m^{-,\uparrow}, u_m^{-,\downarrow}\right)^{\rm T} = \mathbf{U}(u_1, u_2, u_3, u_4)^{\rm T}$. The acoustic Bloch wavefunction is $|m\rangle = u_m^{+,\uparrow}\left|m_{+,\uparrow}\right\rangle + u_m^{-,\uparrow}\left|m_{-,\uparrow}\right\rangle + u_m^{+,\downarrow}\left|m_{+,\downarrow}\right\rangle + u_m^{-,\downarrow}\left|m_{-,\downarrow}\right\rangle$, where $\uparrow\downarrow$ describes the two sublattices in the in-plane honeycomb lattice, and $\pm$ describes the even or odd mirror symmetry of wavefunctions with respect to the plane of $z = h/2$ in Supplementary Fig. 3e. The numerically simulated Bloch wavefunctions of $\left|m_{\pm,\uparrow\downarrow}\right\rangle$ at K point (m = 1) are shown in Supplementary Fig. 4. Since the entire system preserves time-reversal symmetry, the Bloch wavefunctions near K' point ($m = -1$) are a conjugation of those near K point. These Bloch wavefunctions $\left|m_{\pm,\uparrow\downarrow}\right\rangle$ can also be expanded plane-wave basis $|{\rm K}_n\rangle$ (n = 1–3) with three equivalent K points wavevectors

$$\begin{aligned}\left|m_{\pm,\downarrow}\right\rangle &= \frac{e^{i\varphi_{\pm,\downarrow}}}{\sqrt{3}}\sum_{n=1}^{3} e^{i2(n-1)\pi/3} l\left|{\rm K}_n\right\rangle \\ \left|m_{\pm,\uparrow}\right\rangle &= \frac{e^{i\varphi_{\pm,\uparrow}}}{\sqrt{3}}\sum_{n=1}^{3} e^{-i2(n-1)\pi/3} l\left|{\rm K}_n\right\rangle\end{aligned}. \tag{S6}$$

Here $l = \pm 1$ denotes different substructures A and B in Supplementary Fig. 3e. One notes that the additional phase term $\varphi_{\pm,\uparrow\downarrow}$ in these expressions is determined by the specific choice of the five Gamma matrices.

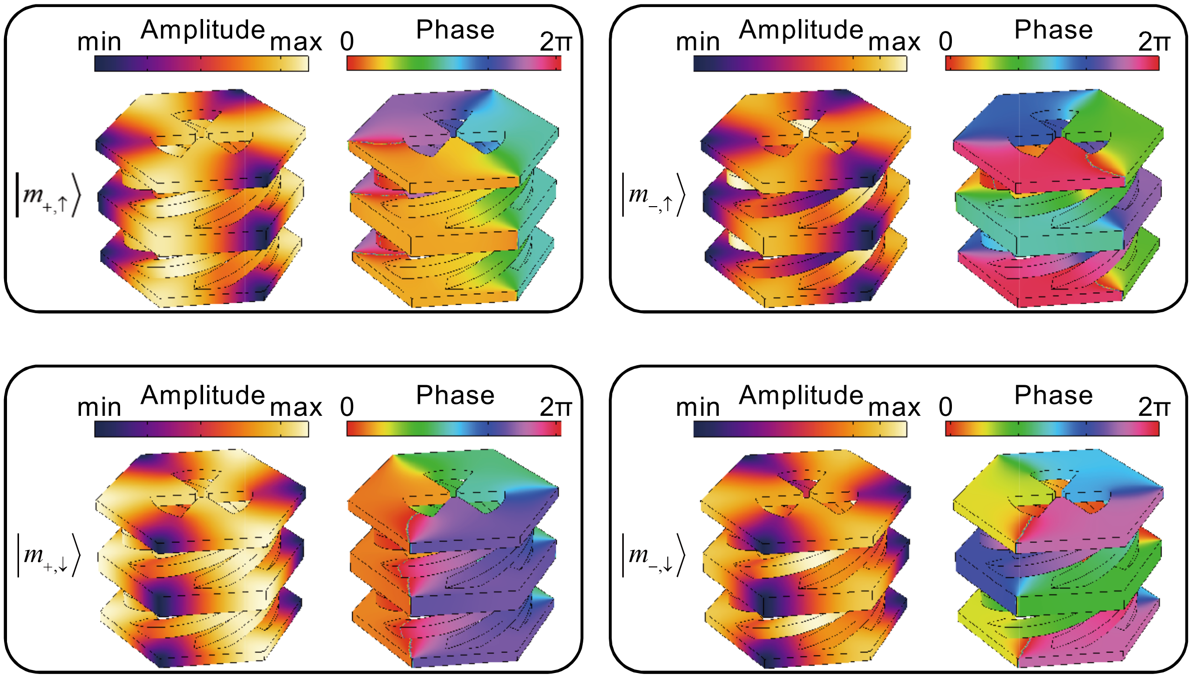

Supplementary Fig. 4 | Simulated Bloch wavefunctions of Yang monopoles at K point.

The eigenvectors of the Yang-monopole Hamiltonian are:

$$\begin{cases} \vec{u}_{1,m} = \left[-i\left(\left|\vec{k}\right| + k_3\right)e^{i\theta} \quad -\left(imk_1 + k_2\right)e^{i\theta} \quad \Delta_0 \quad 0 \right]^{\mathrm{T}} \\ \vec{u}_{2,m} = \left[-i\left(\left|\vec{k}\right| - k_3\right)e^{j\theta} \quad -\left(imk_1 + k_2\right)e^{i\theta} \quad \Delta_0 \quad 0 \right]^{\mathrm{T}} \\ \vec{u}_{3,m} = \left[\left(imk_1 - k_2\right)e^{i\theta} \quad -i\left(\left|\vec{k}\right| - k_3\right)e^{i\theta} \quad 0 \quad \Delta_0 \right]^{\mathrm{T}} \\ \vec{u}_{4,m} = \left[\left(imk_1 - k_2\right)e^{j\theta} \quad -i\left(\left|\vec{k}\right| + k_3\right)e^{i\theta} \quad 0 \quad \Delta_0 \right]^{\mathrm{T}} \end{cases}$$

with corresponding eigenvalues of $(\omega_{\mathrm{YM}} + v_{\mathrm{D}}|\vec{k}|, \omega_{\mathrm{YM}} - v_{\mathrm{D}}|\vec{k}|, \omega_{\mathrm{YM}} + v_{\mathrm{D}}|\vec{k}|, \omega_{\mathrm{YM}} - v_{\mathrm{D}}|\vec{k}|)$.

Here $(k_4, k_5) = \Delta_0 \cdot (\cos\theta, \sin\theta)$ is written in polar coordinates, and $|\vec{k}| = \sqrt{\sum_{n=1}^{5} k_n^2}$ is the norm of the 5D momentum.

Having established the Yang-monopole Hamiltonian around K (K') point, we can calculate the non-Abelian second Chern number of YM by conducting an integration on a 4D manifold surrounding the YM:

$$C_2^{\mathrm{YM}} = \frac{1}{32\pi^2}\int d^4k\varepsilon^{ijkl}\mathrm{tr}\left[f_{ij}f_{kl}\right], \tag{S7}$$

with

$$f_{ij}^{\alpha\beta} = \partial_i a_j^{\alpha\beta} - \partial_j a_i^{\alpha\beta} + i\left[a_i, a_j\right]^{\alpha\beta},$$

$$a_n^{\alpha\beta} = -i\vec{u}_{\alpha,m}^{\mathrm{T}}\frac{\partial}{\partial k_n}\vec{u}_{\beta,m},$$

where $i, j, k, l = 1, 2, 3, 4$. In this equation, ε^{ijkl} is the Levi-Civita symbol, $a_n^{\alpha\beta}$ is a non-Abelian Berry connection and $f_{ij}^{\alpha\beta}$ is the associated non-Abelian field strength. We calculate the second Chern number by conducting integration on the 4D cylinder surface shown in Fig. 1a and obtain that

$$C_2^{\mathrm{YM}} = \frac{1}{2\pi}\int_0^{2\pi} m d\theta = m. \tag{S8}$$

This suggests that the YM located at K and K' points exhibit a second Chern number of 1 and −1, respectively.

1.4. Vortex-string chiral mode

Having analyzed the bulk Hamiltonian of the acoustic metamaterial supporting YM at K and K' points, we now study the vortex-string chiral mode, which is realized by a mapping from the real-space coordinates $(R, \phi, z) = \left[\sqrt{x^2+y^2}, \tan^{-1}(x/y), z\right]$ to the 2D subspace of the 5D space (k_4, k_5):

$$\begin{bmatrix} k_4(\vec{r}) \\ k_5(\vec{r}) \end{bmatrix} = \Delta_0 \begin{bmatrix} \cos(n\cdot\phi+\theta_0) \\ \sin(n\cdot\phi+\theta_0) \end{bmatrix}. \tag{S9}$$

The Hamiltonian in the cylindrical coordinates is:

$$H=\omega_{\mathrm{YM}}+v_{\mathrm{D}}\cdot\begin{bmatrix} k_3 & ime^{im\phi}\left(\frac{\partial}{\partial R}+\frac{im\partial}{R\partial\phi}\right) & -ik_4+k_5 & 0 \\ ime^{-im\phi}\left(\frac{\partial}{\partial R}-\frac{im\partial}{R\partial\phi}\right) & -k_3 & 0 & ik_4-k_5 \\ ik_4+k_5 & 0 & -k_3 & ime^{im\phi}\left(\frac{\partial}{\partial R}+\frac{im\partial}{R\partial\phi}\right) \\ 0 & -ik_4-k_5 & ime^{-im\phi}\left(\frac{\partial}{\partial R}-\frac{im\partial}{R\partial\phi}\right) & k_3 \end{bmatrix} \tag{S10}$$

Here we have assumed that the Fermi velocity is isotropic in all five domains for simplicity. We look for eigenfunctions with the form of $\vec{\psi}=g(R)\cdot[\psi_1,\psi_2,\psi_3,\psi_4]^{\mathrm{T}}$ and eigenvalues $\omega_{\mathrm{YM}}\pm v_{\mathrm{D}}k_3$, where $g(R)$ describe the profile along the radial direction, and $\psi_{1\text{-}4}$ is independent from radial coordinates R. The eigenfunctions can be classified into two groups:

$$\begin{cases} \frac{i\partial\psi_1}{R\partial\phi}=m\frac{\partial g}{g\partial R}\psi_1+\Delta_0(R)e^{i(n+m)\phi+i\theta_0}\psi_4 \\ -\frac{i\partial\psi_4}{R\partial\phi}=m\frac{\partial g}{g\partial R}\psi_4+\Delta_0(R)e^{-i(n+m)\phi-i\theta_0}\psi_1 \end{cases},\omega=\omega_{\mathrm{YM}}+v_{\mathrm{D}}k_3$$

$$\begin{cases} \frac{i\partial\psi_2}{R\partial\phi}=-m\frac{\partial g}{g\partial R}\psi_2+\Delta_0(R)e^{i(n-m)\phi+i\theta_0}\psi_3 \\ -\frac{i\partial\psi_3}{R\partial\phi}=-m\frac{\partial g}{g\partial R}\psi_3+\Delta_0(R)e^{-i(n-m)\phi-i\theta_0}\psi_2 \end{cases},\omega=\omega_{\mathrm{YM}}-v_{\mathrm{D}}k_3$$

For the special case of $m=-1$ (i.e. YM at K' point) and $n=1$ (i.e. $\theta=\phi+\theta_0$), the eigenfunction is:

$$\vec{\psi}=e^{-\Delta_0 R}\begin{pmatrix} e^{i\theta_0/2} & 0 & 0 & e^{-i\theta_0/2} \end{pmatrix}^{\mathrm{T}}.$$

By considering the wavevector along the z direction and applying the approximation that R_0 is nearly zero, we obtain the wavefunction of the VSC mode

$$\psi(R,z)=e^{ik_3 z-\Delta_0 R}\begin{pmatrix} e^{i\theta_0/2} & 0 & 0 & e^{-i\theta_0/2} \end{pmatrix}^{\mathrm{T}},\quad \omega(k_z)=\omega_{\mathrm{YM}}+v_{\mathrm{D}}k_3. \tag{S11}$$

Note that Eq. (S11) operates on Bloch modes $|m_{\pm,\uparrow\downarrow}\rangle$ shown in Supplementary Fig. 4, which can be further expanded into plane wave basis using Eq. (S6), one can obtain the mode profile of the VSC mode:

$$\left|\mathrm{VSC}\right\rangle = \frac{e^{ik_3 z-\Delta_0 R}}{\sqrt{3}} \sum_{n=1}^{3} \left(e^{i\varphi_{+,\uparrow}+i\theta_0/2+i2(n-1)\pi/3} + l \cdot e^{i\varphi_{-,\downarrow}-i\theta_0/2-i2(n-1)\pi/3} \right) \left|\mathrm{K}_n\right\rangle ,$$

which suggests that the component of three K point is no longer equal in the VSC mode if we only look at plane-wave expansions in different sublayers with $l = \pm 1$. If we sum up the total energy distribution of the two sublayers, we can find that the VSC mode has equal energy distribution in the three K points with $\sum_{l=\pm1} \left| e^{i\varphi_{+,\uparrow}+i\theta_0/2+i2(n-1)\pi/3} + l \cdot e^{i\varphi_{-,\downarrow}-i\theta_0/2-i2(n-1)\pi/3} \right|^2 = 4$ independent from for the values of $n = 1 - 3$.

Similarly, for the special case of $m = 1$ (i.e. YM at K point) and $n = 1$ (i.e. $\theta = \phi + \theta_0$), we obtain the wavefunction of VSC mode:

$$\psi\left(R,z\right) = e^{-ik_3 z-\Delta_0 R} \begin{pmatrix} 0 & e^{i\theta_0/2} & e^{-i\theta_0/2} & 0 \end{pmatrix}^{\mathrm{T}}, \quad \omega\left(k_z\right) = \omega_{\mathrm{YM}} + v_{\mathrm{D}} k_3. \tag{S12}$$

Equations (S11) and (S12) represents the topological vortex-string chiral mode related to the two different YMs located at K' and K points, respectively.

In fact, one can always calculate the second Chern numbers of the vortex strings by conducting integration in both the three-momentum axis and the spatial azimuthal direction:

$$\begin{aligned} C_2 &= \frac{1}{32\pi^2} \int_0^{2\pi} d\phi \int d^3 k \varepsilon^{ijkl} \mathrm{tr}\left[f_{ij} f_{kl} \right] \\ &= \frac{1}{2\pi} \int_0^{2\pi} d\phi \frac{d\left[\arg(\Delta)\right]}{d\phi} \\ &= n C_2^{\mathrm{YM}}. \end{aligned} \tag{S13}$$

This suggests that the second Chern numbers of the vortex-string chiral mode are determined by the topological properties of the YMs.

2. Additional experimental results

Figure S5 shows the measured acoustic pressure $p(x,z,f)$ when the source is placed at $z/h = 18$ and 0 during experiments. Specifically, Supplementary Fig. 5a plots the real part of acoustic pressure as a function of frequency and spatial position along z direction. Figure 5b shows the phase term of acoustic pressure as a function of frequency and spatial position along z direction. The frequencies at which the phase term remains constant along the z direction are 4.153 kHz and

4.166 kHz, obtained from forward and backward propagation measurements respectively. The averaged frequency at $k_3 = 0$ is thus 4.16 kHz, displaying a minor deviation from the simulated results of 4.13 kHz in Fig. 1. This discrepancy may be attributed to imperfect sample fabrication or slight deviation in temperatures during the measurements. Note that the relative deviation between the measured and simulated frequency is only 0.72%, indicating a strong agreement between experimental and simulation results. Figure S5c represents the measured spatial distribution of acoustic pressure in the *x*-*z* plane at the frequency of 4.1 kHz. The acoustic waves experience attenuation during propagation. Based on the measured data, we can fit the attenuation factor of the acoustic waves $\alpha(f)$ and subtract the influence of this attenuation factor by plotting normalized acoustic pressure $p(x, z, f) \times e^{z \cdot \alpha(f)}$, which is shown in Fig. 2e in the main manuscript.

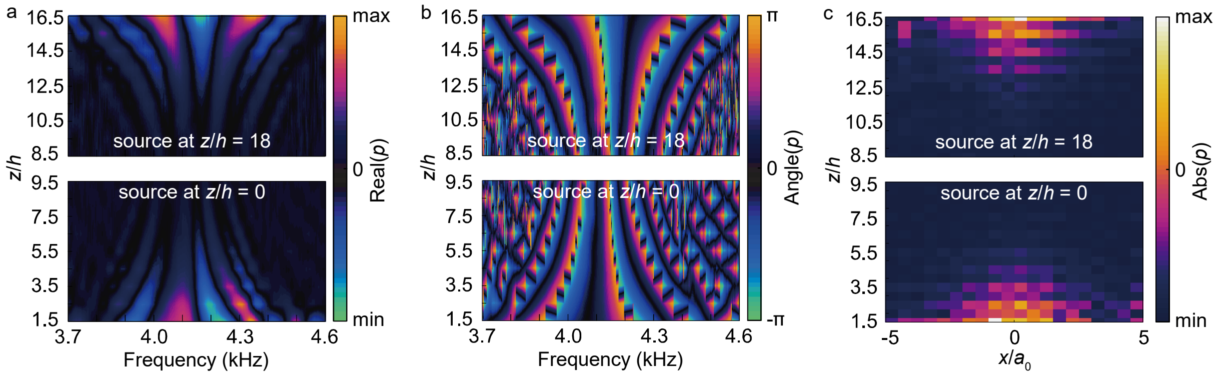

Supplementary Fig. 5 | Measured acoustic pressure *p* when the acoustic source is placed at *z*/*h* = 18 and 0. a, Real part of acoustic pressure as a function of frequency and spatial position along *z* direction. **b,** Phase of acoustic pressure as a function of frequency and spatial position along *z* direction. **c,** Amplitude of acoustic pressure as a function of spatial coordinates *x* and *z* when the acoustic wave is stimulated at 4.1 kHz. One can find that the acoustic waves experience attenuation during propagation along *z* direction.

Supplementary References

S1 Genov, D. A., Zhang, S. & Zhang, X. Mimicking celestial mechanics in metamaterials. *Nat. Phys.* **5**, 687–692 (2009).

S2 Xu, S.-Y. *et al.* Discovery of a Weyl fermion semimetal and topological Fermi arcs. *Science* **349**, 613–617 (2015).

S3 He, C. *et al.* Acoustic analogues of three-dimensional topological insulators. *Nat. Commun.* **11**, 2318 (2020).

S4 Sugawa, S. *et al.* Second Chern number of a quantum-simulated non-Abelian Yang monopole. *Science* **360**, 1429–1434 (2018).
